# Effect of the Stimulus Duration on the Adaptation of the Optokinetic Afternystagmus

**DOI:** 10.3389/fneur.2021.518133

**Published:** 2021-03-31

**Authors:** Jan Gygli, Fausto Romano, Christopher J. Bockisch, Nina Feddermann-Demont, Dominik Straumann, Giovanni Bertolini

**Affiliations:** ^1^Faculty of Medicine, University of Zurich, Zurich, Switzerland; ^2^Department of Neurology, University Hospital Zurich, Zurich, Switzerland; ^3^Swiss Concussion Center, Schulthess Clinic, Zurich, Switzerland; ^4^Departments of Ophthalmology and Otorhinolaryngology, University Hospital Zurich, Zurich, Switzerland

**Keywords:** modeling, ocular motor adaptation, velocity storage mechanism, optokinetic nystagmus, optokinetic afternystagmus

## Abstract

Observing a rotating visual pattern covering a large portion of the visual field induces optokinetic nystagmus (OKN). If the lights are suddenly switched off, optokinetic afternystagmus (OKAN) occurs. OKAN is hypothesized to originate in the velocity storage mechanism (VSM), a central processing network involved in multi-sensory integration. During a sustained visual rotation, the VSM builds up a velocity signal. After the lights are turned off, the VSM discharges slowly, with OKAN as the neurophysiological correlate. It has been reported that the initial afternystagmus in the direction of the preceding stimulus (OKAN-I) can be followed by a reversed one (OKAN-II), which increases with stimulus duration up to 15 min. In 11 healthy adults, we investigated OKAN following optokinetic stimulus lasting 30 s, 3-, 5-, and 10-min. Analysis of slow-phase cumulative eye position and velocity found OKAN-II in only 5/11 participants. Those participants presented it in over 70% of their trials with longer durations, but only in 10% of their 30 s trials. While this confirms that OKAN-II manifests predominantly after sustained stimuli, it suggests that its occurrence is subject-specific. We also did not observe further increases with stimulus duration. Conversely, OKAN-II onset occurred later as stimulus duration increased (*p* = 0.02), while OKAN-II occurrence and peak velocity did not differ between the three longest stimuli. Previous studies on OKAN-I, used negative saturation models to account for OKAN-II. As these approaches have no foundation in the OKAN-II literature, we evaluated if a simplified version of a rigorous model of OKAN adaptation could be used in humans. Slow-phase velocity following the trials with 3-, 5-, and 10-min stimuli was fitted with a sum of two decreasing exponential functions with opposite signs (one for OKAN-I and one for OKAN-II). The model assumes separate mechanisms for OKAN-I, representing VSM discharge, and OKAN-II, described as a slower adaptation phenomenon. Although the fit was qualitatively imperfect, this is not surprising given the limited reliability of OKAN in humans. The estimated adaptation time constant seems comparable to the one describing the reversal of the vestibulo-ocular reflex during sustained rotation, suggesting a possible shared adaptive mechanism.

## Introduction

In healthy individuals, vision remains stable during head and body motion. Stabilization of gaze while moving is achieved through reflexive eye movements that compensate for head motions. These reflexive responses are driven by the integration of several sensory inputs, principally vestibular and visual. While the vestibular system reacts to rapid, high frequency head motions (angular velocity and linear acceleration are directly sensed by the organs in the inner ear), the optokinetic system extracts information on head motion from the observed scene.

The optokinetic system is stimulated by any coherent movement of a large portion of the visual scene. Accordingly, to test the optokinetic system in laboratory conditions, the stimulation is usually performed by horizontally rotating a large, patterned drum around a stationary individual. A person exposed to this stimulation experiences a sensation of self-rotation called circularvection, even though there is no physical motion and no peripheral vestibular stimulation (1, 2). The eyes show a nystagmic response that consists of slow phases drifting in the same direction as the moving stimulus and quick phases in the opposite direction (3).

In humans, as in any foveated animal, both the smooth pursuit system (responsible for maintaining the image of moving target on the fovea) and optokinetic system contribute to the ocular motor response elicited by a large moving pattern (3). Although the whole ocular motor response is usually called optokinetic nystagmus (OKN), the actual response of the optokinetic system is only the reflexive eye movement driven by the coherent motion of large portions of the visual scene on the retina (retinal slip). This mechanism has been described in afoveated animals, where smooth pursuit is not present: OKN builds up slowly, even when the visual stimulus is abruptly accelerated. In humans, however, the eye velocity rises quickly at the beginning of optokinetic stimulation, as the smooth pursuit system rapidly catches up with details of the moving pattern (e.g., stripes of an optokinetic drum). The optokinetic system slowly takes over part of the nystagmic response during sustained stimulations (3). The proportion of the optokinetic system or the smooth pursuit contributing to the gain cannot be established by observing this response only.

An important property of the optokinetic system is the persistence of the response after the stimulus has ended. When an optokinetic stimulus is replaced by total darkness, the nystagmus continues in the same direction. The initial eye velocity reflects the persisting action of both optokinetic systems and smooth pursuit, but the latter is largely gone within 1 or 2 s, causing a characteristic rapid drop of the slow-phase eye velocity (SPEV) of the nystagmus (3). The residual nystagmus, called optokinetic after nystagmus (OKAN) and first described by Ohm (4), continues with a slowly declining SPEV. The initial velocity of OKAN is often measured as soon as smooth pursuit stops and has peak values of around 10 deg/s (5, 6). The rate of decline of OKAN SPEV can be measured fitting the data with a negative exponential curve to determine the time constant. Reported time constant values range considerably, from around 5 s to nearly 50 s (7–11). It is currently accepted that, during the stimulation period, the optokinetic system effectively acts through the velocity storage mechanism (VSM) (3). The VSM is a central integrative network that plays a role in the integration of multisensory rotational stimuli (12, 13) and presumably involves the medial and superior vestibular nuclei (3, 14–17). The VSM can be regarded as a neural integrator that receives input from the vestibular and visual systems and controls the slow dynamics and the compensatory eye velocity of the vestibular and optokinetic reflexes. The OKAN response is considered to be a direct manifestation of the VSM integrator (12, 18), where the decay rate of SPEV is controlled by the time constant of the VSM integrator (high value of the time constant indicates slow decay in SPEV). The relation between OKAN and vestibular nystagmus through VSM was confirmed by animal studies: In monkeys the above-mentioned vestibular nuclei neurons that respond to head rotation are also stimulated by optokinetic stimuli. When the lights are turned off after optokinetic stimulation, the vestibular nucleus neurons continue discharging for some more seconds; this is the neurophysiological correlate for OKAN (3, 19).

In some individuals and in specific conditions OKAN SPEV does not simply decline to zero, but it changes direction and, accordingly, a secondary afternystagmus (named negative OKAN or OKAN-II) beating in the opposite direction is observed. The OKAN-II has been reported in both human adults (11, 20), infants (21), and in different species, including monkeys (22, 23), rabbits (24–26), cats (27, 28) and rats (29).

There are different theories on how OKAN-II is generated. A general hypothesis has been proposed by Brandt et al. (20). They suggested that the optomotor response is determined by two opposite tendencies: the rapidly fading positive optokinetic charge and the much longer lasting habituative countercharge. As long as the first outweighs the second, positive OKAN (OKAN-I) is observed. At an intermediate period, the two mechanisms may balance each other (nystagmus disappears) until with further decay of positive charge the counter regulation determines the onset of OKAN-II (20). In the same study it was also suggested that prolonged optokinetic stimulation may cause ocular motor and perceptual aftereffects similar to those observed on per-rotatory or post-rotatory nystagmus (20). They also reported that background movement of the whole visual field when the eyes are fixed on a stationary target in the foreground facilitates occurrence of an observable OKAN-II (20). This evidence was viewed as a possible indication that the OKAN-II is caused by a central nervous adaptation driven by the motion of the visual field on the retina during a period of stimulation, suggested by Chen et al. and by König et al. (30, 31). On the contrary, it has been proposed that OKAN-II may be related to ocular motor adaptation induced by continued eye tracking. This was shown in a patient with Infantile Nystagmus Syndrome (INS), who lacks a normal smooth-pursuit function, and therefore cannot follow the stripes. The afternystagmus of the patient in darkness was beating in the direction opposite to the preceding OKN and was interpreted as an unmasked negative OKAN. This interpretation assumes that, in healthy controls, the negative OKAN is probably masked by the afternystagmus of smooth pursuit (32). These hypotheses were not formalized in mathematical models or diagrams. A different approach was presented by Furman et al. (33). They adapted established VOR-OKAN models to account for a reversal SPEV showing that both a peripheral adaptation operator and a central adaptation operator can account for OKAN-II. A similar reversal also occurs during vestibular nystagmus in response to sustained angular rotation (34, 35).

Although OKAN-II has been reported in humans, quantitative information specifying occurrence, strength and time dynamic are scarce and contrasting. This is not surprising as OKAN itself is known to be weaker in humans than in other species and thus difficult to quantify consistently. Koenig and Dichgans (31) reported no OKAN-II in four humans tested with optokinetic stimuli at 30, 60, 90, 120, and 180°/s for 1 min. Brantberg (36), who defined OKAN-II if the SPEV inversion reached 2°/s, reported occurrence of OKAN-II in 3 out of 256 trials in 16 participants (optokinetic stimuli at 60 and 90°/s for 1 min). Interestingly all instances of OKAN-II were observed in the same participant. Nooji et al. (11), using the same criteria and a stimulus velocity at 60°/s, described it in 4 out of 13 participants. In contrast, data presented by Brandt et al. (20) suggest that OKAN-II is highly present in humans already after 1 min of optokinetic stimulation. Their data also demonstrated a dependency of both OKAN-I and OKAN-II on the duration of the optokinetic stimuli. Although they present the most extensive description of OKAN-II in humans, only mean values of total amplitude were provided and no dispersion metrics or details of occurrence of OKAN-II across participants were provided. Although other studies (7, 37) mentioned occurrence of OKAN-II in humans, quantitative observation are missing.

Understanding the specific of OKAN-II in humans can be important to evaluate the potential impact of the OKAN-II on the assessment of OKAN-I. While, as of now, OKAN has no direct diagnostic use, a clinical potential has been discussed in many studies (38–44).

Understanding OKAN-II is also important to design studies that could allow to evaluate applicability of the model of Furman et al. (33) in humans, including extension to perceptual aftereffects and adaptation to prolonged optokinetic stimuli. This is particularly relevant considering re-emerging interest in OKAN with respect to investigating vection in virtual reality and the potential risk for visually induced motion sickness (11, 37). Recent studies, either entirely neglected OKAN-II (37) or used descriptive models without a physiological background (7, 11). Both approaches may risk a misestimation of OKAN-I parameters too (34). A parsimonious model that could be descriptive of human data while accounting for the considerations and the evidence summarized by Furman et al. (33) is indeed missing.

The current study aimed to objectively quantify the dependency of the OKAN-II on the duration of the preceding optokinetic stimulation and to describe its occurrence and main features. In addition, we evaluated how a simple descriptive model, which considers OKAN-I and OKAN-II to be two independent phenomena could account for OKAN-I and OKAN-II changes across duration.

## Materials and Methods

### Participants

Nineteen healthy human participants (7 males, 12 females, mean age 24 years, range 19–36 years) participated in the study. Informed consent of all participants was obtained in written form after full explanation of the experimental procedures. The protocol was approved by the ethics Committee of the Canton of Zurich, Switzerland (Protocol N° KEK-ZH-2012-0150) and was in accordance with the ethical standards laid down in the 2013 Declaration of Helsinki for research involving human participants.

### Experimental Setup

Participants were comfortably seated upright on a chair mounted on a two servo-controlled motor-driven axes turntable system (Tönnies D561, Freiburg i.Br., Germany; control system: Acutrol® ACT2000, Acutronic, Switzerland Ltd.). The two, independent motor-driven axes are coincident and earth vertical. One rotates the chair and the other a cylinder (Optokinetik Drum, radius: 74 cm) mounted concentrically to the chair. Remotely controlled LEDS are attached to the cylinder at the level of participant's eyes. Safety belts around the feet and the shoulders restrained the participant. An adjustable chin rest and a forehead strap were used to stabilize the participant's head.

### Recording of Eye Movements

Horizontal eye movements were recorded at 220 Hz with a head-mounted video-oculography (VOG) device (“EyeSeeCam”) (45) consisting of goggles with one mounted infrared camera on the left eye. A model of the eye rotation is used by the VOG system to derive the horizontal eye position from the pupil position recorded in the coordinate system of the cameras. An additional offline calibration was performed to improve the accuracy. Before the beginning of the experiment, participants were asked to look at a grid of fixation points according to a sequence generated using the LED attached to the motorized cylinder. A second order polynomial function was fitted to the corresponding eye angles provided by the VOG system for calibration as in Bertolini et al. (46).

### Optokinetic Stimulus

The inner wall of the drum was covered with 10 cm alternating black and white stripes, each subtending 7.7 deg of visual angle. The pattern filled the entire visual field including the retinal periphery. A small gray band at eye level, extending 8 deg vertically, was overlapped on the stripes. It served as reference for gaze to minimize foveal fixation.

### Experimental Procedure

In all experiments, the optokinetic drum was accelerated in darkness to 30 deg/s about the earth-vertical axis while the chair was kept stationary. The lights inside the drum were suddenly turned on and the tested participant was asked to keep the gaze on the gray panel without fixating. After a fixed time (30 s, 3 min, 5 min, and 10 min), the light was switched off to terminate the optokinetic stimulation, but the recording of eye movement continued until no nystagmus could be observed. To ensure that the participants remain focused and compliant during all stimuli, the examiner controlled the eye video and position trace in real time and encouraged them in case changes in the nystagmus behavior (or other signs of distraction) occurred.

The experimental procedure was divided in two subsequent phases. All 19 participants took part in the first phase. The drum was rotated at 30 deg/s for 30 s, inducing OKN. Two trials were performed with the drum rotating in clockwise direction and two in counterclockwise direction.

After the first phase, the OKN was analyzed to evaluate the quality of the participants' responses to optokinetic stimuli. Exclusion criteria were bad adherence (e.g., due to lack of concentration during the experiment), calibration problems and noisy data (e.g., due to repetitive blinking). Using these criteria, 11 participants were selected for the second phase. As no asymmetry is reported in normal participants (8), phase two was based on testing OKN in a single direction. Based on the results of phase one, a favorable direction was selected for each participant as the one with the stronger, more consistent and less noisy OKAN response. In the experiment of the second phase, the drum rotated at 30 deg/s in the participant's favorable direction for 3, 5, and 10 min, inducing OKN and vection. Two trials were performed for each duration and the sequence of the experiment was randomized. The trials were separated by a pause lasting a minimum of 60 s with the lights on and the drum not moving, allowing the discharge of any potential velocity storage activity.

### OKAN Analysis

Only eye movements recorded after the light was terminated were considered in the analysis. These eye movement signals are expected to represent three phenomena: the OKAN-I, the OKAN-II and the rapid drop of smooth-pursuit. To obtain the SPEV, eye velocity was calculated as the derivative of eye position and saccades were removed using a median filter with a 2 s time window (MatLab function: medfilt1). To address the rapid decay of the smooth pursuit, we excluded the first 2 s of eye movements after the light was turned off from all subsequent analysis. The sign of the eye movement signal was adjusted so that the OKN SPEV was positive. This implies that OKAN-I has a positive sign and OKAN-II a negative sign in all trials. Three independent analyses were performed: area-based analysis, SPEV analysis and model-based analysis.

#### Area-Based Analysis

The aim of this analysis was to quantitatively measure the occurrence of OKAN-II and to determine the time of transition between OKAN-I and OKAN-II (subsequently named OKAN-II onset). The cumulative area under the SPEV curve (47) was calculated for the first 60 s after light termination. A positive peak in the cumulative area under the SPEV implies that the SPEV crossed zero (i.e., a sustained positive SPEV followed by a sustained negative SPEV causes an increase of the cumulative area followed by a decrease). The first positive peak after which the cumulative area decreased steadily for 10 s was considered as OKAN-II onset. This allowed to discard peaks caused by zero-crossings due to noise. The occurrence of OKAN-II and its onset were evaluated within and between participants and as function of stimulus duration.

#### SPEV Analysis

The aim of this analysis was to quantify how SPEV was affected by stimulus duration during OKAN-I and OKAN-II. For OKAN-I the maximal value of positive SPEV before the OKAN-II onset (as identified according to the area-based analysis) and discarding the first 2 s (to account for smooth pursuit) was used as peak velocity. OKAN-II peak velocity was calculated as the minimum value of negative SPEV after the OKAN-II onset. This analysis was constrained to the first 60 s to allow a comparison with previously published OKAN-II descriptions (11, 36). In these studies, the occurrence of OKAN-II was defined as a SPEV passing a threshold of −2 deg/s, with the shortest OKAN recordings lasting 60 s.

#### Model-Based Analysis

The aim of this analysis was to analyze fit parameters using a simple descriptive model accounting for OKAN-II. In the majority of the OKAN studies, the following exponential function (12) was used to fit the SPEV *V*(*t*):

(1) $$\begin{array}{l} V\left(t\right)=\;a\;\cdot e^{-t/\tau_{1}} \end{array}$$

In Equation (1), *a* is the initial amplitude of the OKAN-I SPEV and τ_1_ the time constant of the OKAN-I. OKAN-II, however, is not accounted for. As the main aim of the current study is to describe OKAN-II and its relation to OKAN-I, we added to Equation (1) an additional independent exponential term with an amplitude parameter having the sign opposite to the optokinetic stimuli. The OKAN SPEV *f*(*t*) was fitted using the following double exponential function:

(2) $$\begin{array}{l} f\left(t\right)=\;a\;\cdot e^{-t/\tau_{1}}-b\;\cdot \;e^{-t/\tau_{2}} \end{array}$$

In Equation (2), *a* reflects the initial amplitude of the OKAN-I SPEV, *b* the initial amplitude of the OKAN-II SPEV, τ_1_ the time constant of the OKAN-I and τ_2_ the time constant of the OKAN-II. This approach can be seen as simplified version of the model suggested by Furman et al. (33), where peripheral and central adaptation elements are combined with a velocity storage model to generate a second-order system with damping. If the damping ratio is larger than one, a condition corresponding to what the authors defined as reproducing the normal vestibulo-ocular reflex response to step input, the equation proposed by Furman et al. for the OKAN SPEV is equivalent to Equation (2) [see Appendix of Furman et al. (33)]. Importantly this choice differs from the one of Laurens et al. (7), who used a saturation model toward a negative asymptote.

**Figure 1:**
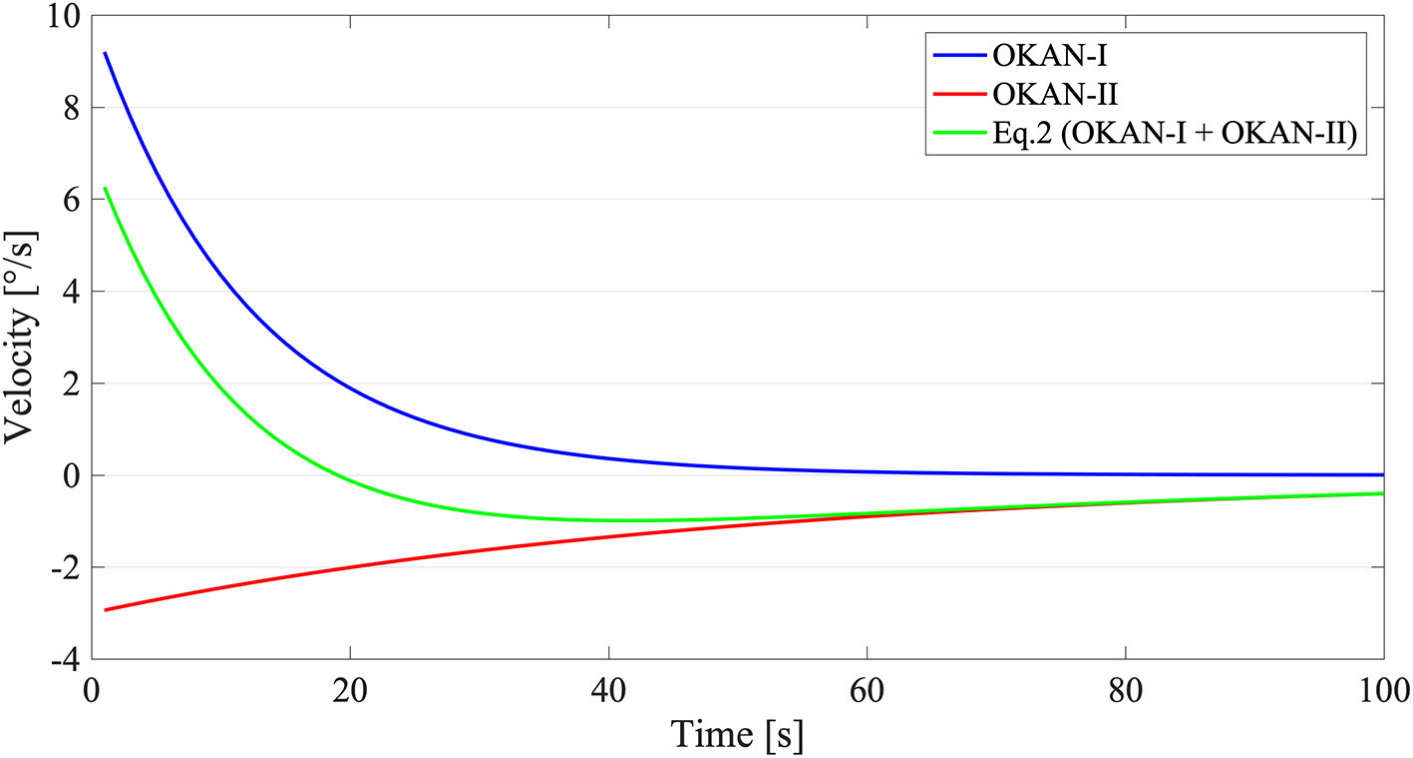
Graphical Explanation of Equation (2). The blue line stands for OKAN-I ($a\;\cdot e^{-t/\tau_{1}}$) with an initial velocity of 10 deg/s and a time constant of 12 s. The red line on contrary symbolizes OKAN-II ($-b\;\cdot \;e^{-t/\tau_{2}}$) with an initial velocity of 3 deg/s and a time constant of 50 s. The green line corresponds with Equation (2) ($f\left(t\right)=\;a\;\cdot e^{-t/\tau_{1}}-b\;\cdot \;e^{-t/\tau_{2}}$) where both OKAN-I and OKAN-II are accounted for.

To reduce the risk of overfitting, we assumed that the OKAN-I term is independent from the stimulus duration (after its initial charging time <30 s) (13) and that OKAN-II contribution before 30 s of stimulation is minimal (20). Accordingly, in a first step, we fitted the response to the trial with 30 s of optokinetic stimuli with Equation (1) and, subsequently, we used the value of *a* (initial amplitude of OKAN-I) and τ_1_ (time constant of OKAN-I) estimated from the 30 s trials to fit Equation (2) on all remaining trials (Figure 1). The parameters *a* and *b* were bound between 0 and 20 deg/s, τ_1_ between 1 and 40 s and τ_2_ between 1 and 150 s. These boundaries are in line with previous data on OKAN (8, 10, 11) and secondary nystagmus in vestibuloocular reflex (VOR) (7, 48).

### Statistical Analysis

Statistical testing was performed in MATLAB. The chi-square-test for proportions was used to compare occurrence of OKAN-II at the different stimulus durations. All other continuous variables (i.e., onset time, peak velocity, time constants) were analyzed depending on the number of groups to compare. Specifically, the paired *t*-test was used when only two groups were compared, while a repeated-measures ANOVA for multiple groups. When *post-hoc* multiple comparisons were performed, Tukey's honest significant difference procedure was used to retain family-wise error rate. For all tests, we considered significant a *p*-value < 0.05 after multiple comparison correction.

## Results

According to the exclusion criteria (e.g., noisy data due to frequent blinking or bad adherence) data of 8 of the 19 participants were discarded from further testing after the acquisition of the 30 s condition. Of the remaining 11 participants, 4 out of 88 trials were discarded as the participant asked for early termination of the trial or did not maintain a stable OKN during stimulation.

**Figure 2:**
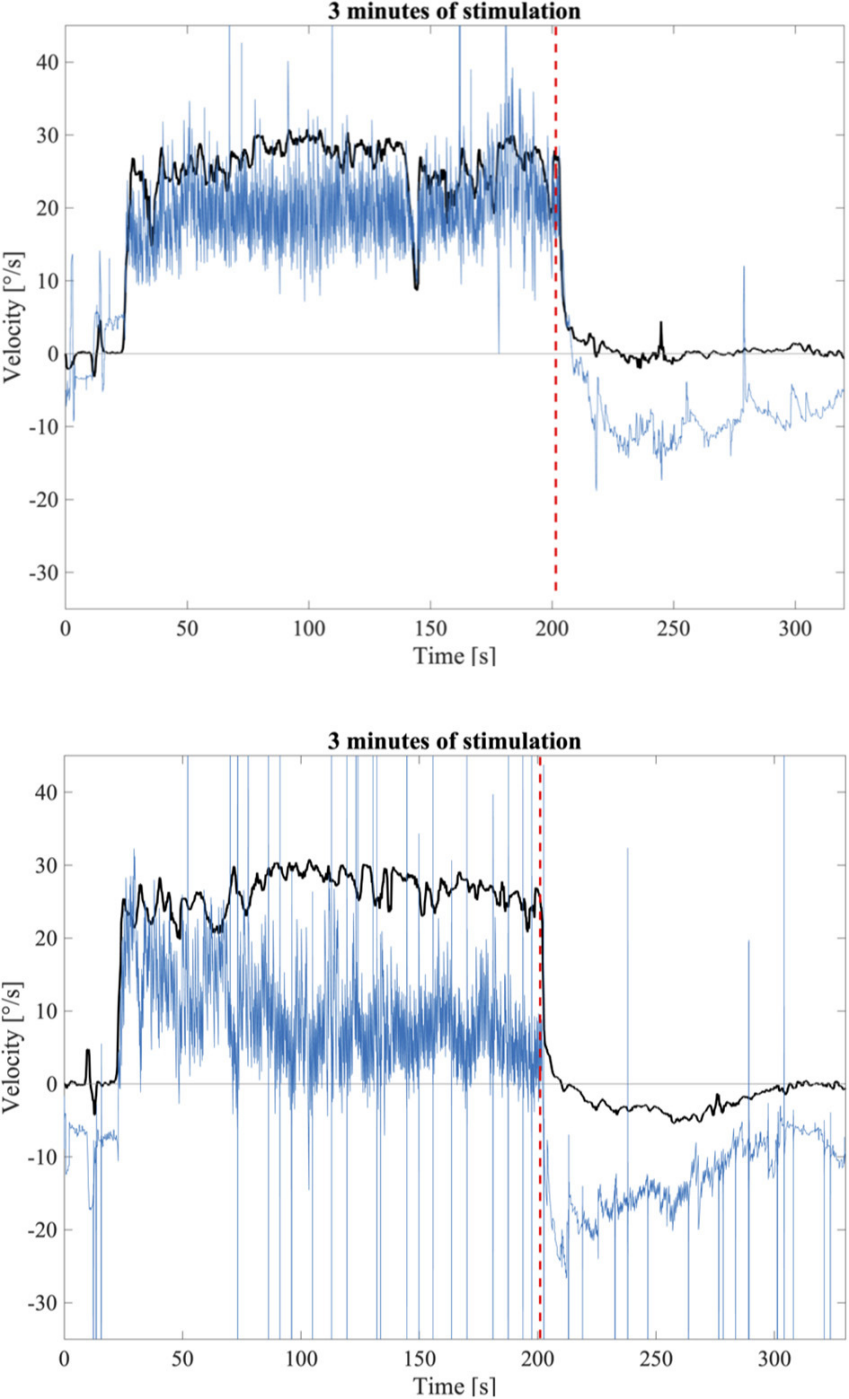
**Top:** Example of a SPEV response (Black) and the eye position trace (Blue) to 3 min of optokinetic stimuli without OKAN-II; **Bottom:** Example of a SPEV response (Black) and the eye position trace (Blue) to 3 min optokinetic stimuli with OKAN-II that could be identified by visual inspection. The dashed red line indicates the instant where the light was terminated.

Visual inspection of the responses to the longer stimulation confirmed the presence of OKAN-II. The occurrence of OKAN-II, however, varied considerably between participants. Examples of slow phase eye velocity in two participants, one without OKAN-II and one showing a typical OKAN-II following 3 min of stimulation are shown in Figure 2. In both cases, after turning the light off the SPEV suddenly dropped, matching the prediction of rapidly fading smooth pursuit contribution. Subsequently, the residual SPEV followed an exponential decay, known as OKAN-I. The OKAN-II is observable in the bottom graph: the SPEV reaches zero earlier than in the top graph, becomes negative (i.e., the nystagmus is now beating in the opposite direction) and continues with a negative sign for a period of time, returning to zero with a decay slower the one observed for OKAN-I.

### Occurrence, Onset Time, Peak Velocity

Visual inspection of the OKAN traces showed that OKAN-II could be observed in 3 of the 11 participants, accounting for 27% of the participants. Pooling all trials (participants and durations) and considering OKAN-II present when the cumulative area under the SPEV decreases for at least 10 s (i.e., consistent negative SPEV for 10 s), 25 out of 84 analyzed trials (30%) could be identified as OKAN-II. The occurrence of OKAN-II divided per stimulus duration and per participant is presented in Table 1. Notably only 5 participants had more than 1 trial showing OKAN-II. They account for 23/25 (92%) trial with OKAN-II.

**Table 1:** OKAN-II incidence.

		**Stimulus duration**				
		**30 s**	**3 min**	**5 min**	**10 min**	**Tot. per participant**
Participants	S01	0/2	1/2	0/2	0/2	1/8
	S02	1/2	2/2	2/2	2/2	7/8
	S03	0/2	1/2	2/2	1/2	4/8
	S04	0/2	2/2	1/2	1/2	4/8
	S05	0/2	0/2	0/2	0/2	0/8
	S06	0/2	0/2	0/2	0/2	0/8
	S07	0/2	0/2	0/2	0/1	0/7
	S08	1/2	0/2	0/2	0/2	1/8
	S09	0/2	2/2	1/2	1/2	4/8
	S10	0/2	0/1	0/1	0/1	0/5
	S11	0/2	2/2	1/2	1/2	4/8
	Tot. per duration	2/22	10/21	7/21	6/20	**Total:** 25/84

*Green: subjects with consistent OKAN-II occurrence. Yellow: subjects with sporadic (1 out of 8 trials) OKAN-II occurrence. Blue: subjects without OKAN-II occurrence*.

Using the criteria proposed by Brantberg (36), only 11 of 84 analyzed trials (13%) showed OKAN-II, with 10/11 OKAN-II trials recorded from 3 of the 5 participants identified above. Of these, only two participants had more than one OKAN-II trial and accounted for 9/11 OKAN-II trials (82%).

Considering that only 2/22 trials (9%) with 30 s of optokinetic stimulus showed OKAN-II, the analysis was focused on the trials with the longer stimulus durations and limited to the 5 participants consistently showing OKAN-II. This selection accounts for 22/25 (88%) of the OKAN-II trials observed in the whole study. Occurrence, onset time and peak velocity of OKAN-II in these trials are detailed in Tables 2A, 2B. A significant effect of the stimulus duration was found for the onset time of OKAN-II [repeated measure ANOVA; *F*_(2,8)_ = 6.63, *p* = 0.02], *post-hoc* analysis showed that onset-time after 10 min is significantly later than after 3 min (*p* = 0.014), while no difference was found between 3 and 5 min (*p* = 0.89) and 5 and 10 min (*p* = 0.17). No effect was found for OKAN-II occurrence (Chi-square = 2.39, *p* = 0.30), for the peak positive SPEV during OKAN-I [repeated measure ANOVA; *F*_(2,8)_ = 0.84, *p* = 0.46] and the peak negative SPEV during OKAN-II [repeated measure ANOVA; *F*_(2,8)_ = 0.64, *p* = 0.51].

**Table 2A:** OKAN-II incidence.

		**Stimulus duration**			
		**3 min**	**5 min**	**10 min**	**Tot. (per participant)**
Participants	S02	2/2	2/2	2/2	6/6
	S03	1/2	2/2	1/2	4/6
	S04	2/2	1/2	1/2	4/6
	S09	2/2	1/2	1/2	4/6
	S11	2/2	1/2	1/2	4/6
	Tot. (per duration)	9/10	7/10	6/10	**Total:** 22/30

*Green: subjects with consistent OKAN-II occurrence. Yellow: subjects with sporadic (1 out of 8 trials) OKAN-II occurrence. Blue: subjects without OKAN-II occurrence*.

**Table 2B:** OKAN-II onset time (s) and peak velocity (deg/s).

		**Onset time (s)**				**Peak velocity (deg/s)**			
		**Stimulus duration**			**Mean ± SD (per participant)**	**Stimulus duration**			**Mean ± SD (per participant)**
		**3 min**	**5 min**	**10 min**		**3 min**	**5 min**	**10 min**	
Participants	S02	2.05	5.55	5.3	4.30 ± 1.95	−2.64	−1.87	−2.85	−2.45 ± 0.51
	S03	6.23	12.72	15.07	11.34 ± 4.58	−1.24	−1.31	−0.71	−1.09 ± 0.33
	S04	28.33	20.42	37.19	28.65 ± 8.39	−1.97	−1.57	−1.97	−1.83 ± 0.23
	S09	8.17	6.97	22.35	12.50 ± 8.56	−3.39	−4.9	−1.98	−3.43 ± 1.46
	S11	21.9	26.99	32.06	26.98 ± 5.08	−1.39	−1.15	−0.71	−1.08 ± 0.34
	Mean ± SD (per duration)	13.34 ± 11.21	14.53 ± 9.10	22.40 ± 12.83	**Mean** **±** **SD:** 16.75 ± 11.13	−2.13 ± 0.90	−2.16 ± 1.56	−1.64 ± 0.92	**Mean** **±** **SD:** −1.98 ± 1.11

*Green: subjects with consistent OKAN-II occurrence. Yellow: subjects with sporadic (1 out of 8 trials) OKAN-II occurrence. Blue: subjects without OKAN-II occurrence*.

While the peak negative SPEV during OKAN-II could only be calculated when OKAN-II was identified, the peak positive SPEV during OKAN-I could be calculated in all trials. Focusing on the five participants consistently showing OKAN-II, this value was significantly larger (paired *t*-test; *p* = 0.03) in the 30 s trials than in the pooled trials from all other durations. This effect was not observed in the six participants not showing OKAN-II (paired *t*-test; *p* = 0.63) (Tables 3A, 3B).

**Table 3A:** OKAN-I peak velocity (deg/s): participants with OKAN-II.

		**Stimulus duration**			**Comparison**	
		**3 min**	**5 min**	**10 min**	**30 s**	**Mean of all trials with longer durations**
Participants	S02	1.79	2.13	2.6	4.17	2.17
	S03	5.63	5.43	4.64	7.20	5.19
	S04	4.11	3.58	3.46	3.66	3.64
	S09	5.03	6.9	8.27	10.71	7.07
	S11	5.48	5.56	6.57	6.98	5.95
	Mean ± SD (per duration)	4.41 ± 1.41	4.72 ± 1.67	5.11 ± 2.07	6.54 ± 2.83	4.96 ± 2.19

*Green: subjects with consistent OKAN-II occurrence. Yellow: subjects with sporadic (1 out of 8 trials) OKAN-II occurrence. Blue: subjects without OKAN-II occurrence*.

**Table 3B:** OKAN-I peak velocity (deg/s): participants without OKAN-II.

		**Stimulus duration**			**Comparison**	
		**3 min**	**5 min**	**10 min**	**30 s**	**Mean of all trials with longer durations**
Participants	S01	6.01	7.73	6.51	6.03	6.56
	S05	8.56	12.81	11.47	7.89	10.95
	S06	5.66	6.08	8.29	6.67	6.67
	S07	6.45	4.80	8.40	10.23	6.55
	S08	7.43	7.85	9.15	4.27	8.14
	S10	7.33	9.64	5.51	7.27	7.49
	Mean ± SD (per duration)	6.91 ± 1.07	8.15 ± 2.82	8.22 ± 2.08	7.06 ± 1.99	9.63 ± 1.98

*Green: subjects with consistent OKAN-II occurrence. Yellow: subjects with sporadic (1 out of 8 trials) OKAN-II occurrence. Blue: subjects without OKAN-II occurrence*.

### Model Fit

Considering all participants, the mean time constant of OKAN-I estimated using Equation (1) to fit the response to 30 s of optokinetic stimulus was 18.5 ± 10.1 s (Mean ± SD). The mean initial velocity of OKAN-I estimated using Equation (1) was 6.5 ± 1.9 deg/s.

**Figure 3:**
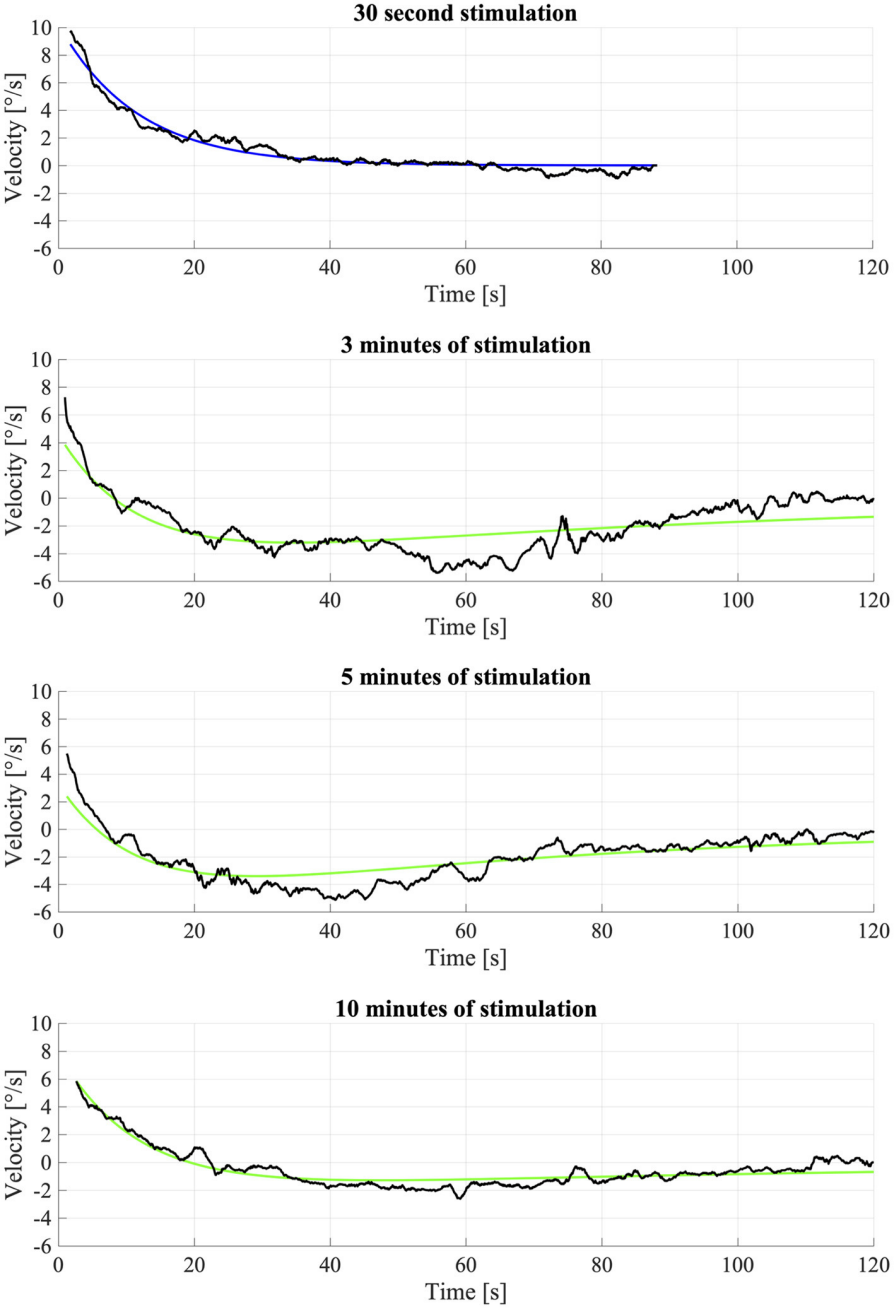
Fitting of OKAN-II S09. The fitting of the 30 s condition was used for fixing the OKAN-I parameters for the other (3, 5, 10 min) conditions and is therefore fitted with Equation (1). The longer conditions (3, 5, 10 min) were fitted with Equation (2). Due to noise and inconsistency the fit wasn't particularly successful.

As the data analysis above evidenced that OKAN-II occurred consistently in only 5 participants, we performed the analysis using Equation (2) on their data only. The model failed to fit the data of one participant (S03), which was discarded from the analysis. No significant difference between the response to the longer optokinetic stimuli (3, 5, 10 min) was found for the time constants [repeated measure ANOVA; *F*_(2,2)_ = 0.90, *p* = 0.53] or the amplitude of OKAN-II [repeated measure ANOVA; *F*_(2,6)_ = 2.87, *p* = 0.13]. The mean time constant for 3 min was 104.8 ± 38.7 s, for 5 min 104.6 ± 60.8 s, and for 10 min 71.8 ± 57.0 s. The mean amplitude 2.9 ± 2.2 deg/s, 2.1 ± 2.4 deg/s, and 1.3 ± 1.3 deg/s for the 3, 5, and 10 min trial, respectively (Figure 3).

## Discussion

The results of the current study confirm that a prolonged optokinetic stimulus (longer than 3 min in our experimental design) increases the probability for OKAN-II to appear, as reported by previous studies (11, 20, 30). OKAN-II follows the decaying OKAN-I; the slow-phase eye velocity (SPEV) of OKAN-II is directed opposite to the direction of the preceding optokinetic stimulation and the hence the direction of SPEV of OKAN-I.

Although the incidence OKAN-II was relatively low in our study (only 5/11 of our participants showed it in more than one trial), but higher than previously reported [Brantberg (36) 1/16 participants; Nooji (11) 4/13 participants]. This may depend on the criteria used for defining the occurrence of OKAN-II. The two aforementioned studies used the criteria defined by Brantberg (36), i.e., a SPEV exceeding 2 deg/s in the direction opposite to the preceding OKAN. Regarding the noise and variability in human OKAN responses, we considered such threshold quite high and possibly specific for the properties (e.g., velocity) of the stimulus and used a different method. As previously suggested by Hain et al. (47), the impact of noise on OKAN can be reduced by analyzing cumulative SPEV. We thus defined that any trial where the cumulative SPEV decreased for at least 10 s (i.e., consistent negative SPEV for 10 s) after reaching a peak contained OKAN-II. Although 10 s are also an arbitrary threshold, it is well below the expected time constant of the adaptation process underlying OKAN-II (34, 35). Thus, it seems safe to assume that OKAN-II, if occurring, should last more than 10 s.

The incidence analysis (Tables 1, 2A, 2B) suggests that OKAN-II occurrence may be subject-specific in humans. Participants who showed OKAN-II at least twice (5/11 participants), tended to have it quite consistently, i.e., in over 70% of their trials with stimulus duration of 3 min or more (24/30 trials). The other participants almost never showed it (2/44 trials−4%). Evidence supporting our observation can be found in previous studies. Brantberg (36) found OKAN-II in 1 out of 16 participants, but the participant showed it 3 times. Nooij (11) reported it in 24% of all recorded trials but only in 4 participants. As 4 participants in a population of 13 participants account for roughly 31% of the total data, the mentioned 24% imply that OKAN-II was consistently shown in over 75% of the trials of the 4 participants. The instants of reversal of SPEV (onset of OKAN-II) may also be subject-specific, as suggested by the comparison of the standard deviations within participant (row) and between participants (columns) in Table 2B.

Brandt et al. (20) proposed that the strength of the OKAN-II response continues to increase with duration of stimulation up to 15 min. As none of the features we calculated support an increase of the OKAN-II with stimulus duration longer than 3 min, we could not confirm this observation. On the contrary, we found that OKAN-II onset appeared later with stimulus duration of 10 min when compared to 3 min suggesting that OKAN-II onset time may occur later after longer stimulation. Assuming OKAN-I (amplitude and/or time constant) does not increase with these stimulus durations from 3 to 10 min, our finding would suggest a decrease of OKAN-II (amplitude and/or time constant), which is unlikely for a simple adaptation phenomenon. As OKAN-II has been described as part of an oscillatory behavior of the SPEV (33), the later appearance of onset with stimulus duration may be due to effects not accounted in this study. The differences between our observations and those of Brandt et al. (20) are probably best explained by the different analysis methods. Brandt et al. split OKAN-I and OKAN-II based on the sign of the overall resulting velocity, a method that is sensitive to noise in the data, and evaluated the total amplitude of the nystagmus, a measure that critically depends on the overall time evaluated. Although we also used eye position in the analysis (cumulative SPEV to define OKAN-I and OKAN-II), we analyzed specific features that did not depend on the area (onset time, peak velocity).

To evaluate the time course of both OKAN-I and OKAN-II as a function of the stimulus duration, we propose a simple model-based approach. The OKAN responses observed in our experiments using short optokinetic stimuli (30 s) were characterized by a SPEV in the same direction of the previous OKN that decay to zero as a single exponential. This behavior matches the expected OKAN behavior extensively described by previous studies describing the OKAN as a manifestation of the velocity storage contribution to the OKN (12). Accordingly, the response to the 30 s stimuli was well-captured by a single exponential model with two parameters: the time constant of the exponential function (8, 12) and the initial velocity amplitude. Our values were in agreement with those measured in earlier studies (5, 7, 8, 10, 11, 37, 47).

When responses showed OKAN-II, however, a single exponential accounting for OKAN-I is misleading. It has been shown previously for VOR that errors in the estimates of the time constant of the primary nystagmus occur when the secondary nystagmus is not accounted for (34). In our study we adopted a model (Equation 2) that considers OKAN-I and OKAN-II as independent decaying exponential functions with opposite sign, starting from the moment the optokinetic stimulus is terminated. Our model can be viewed as a simplified version of the model proposed by Furman et al. (33).

Even though the model was not able to follow the responses in all fitted trials of the subjects presenting OKAN-II [*r*^2^ range (0.3–0.8); mean ± SD = 0.5 ± 0.2], the validity of Equation (2) (i.e., a simplified model) should be considered in the context of modeling OKAN-II in humans. Descriptions of OKAN-II in humans are lacking with only a few studies actually mentioning it (7, 11, 20, 36), its incidence is low, its variability elevated and its analysis challenging (47) compared with other mammals such as monkeys (19). In contrast rigorous OKAN models accounting for OKAN-II (20, 33, 36) propose an order of complexity that may be overfitting the weak, noisy response available in humans.

To solve this problem Nooij et al. and Laurens et al. decided to fit OKAN-II in humans with the following equation suggested for the VOR (7, 11):

(3) $$\begin{array}{l} V\left(t\right)=\;-m\;\cdot \left(1-e^{-t/\tau_{2}}\right) \end{array}$$

The rate of decrease is governed by the time constant τ_2_ and the maximum value is determined by the parameter m. Thus Equation (3) represents a saturation model, which contrast with the current hypothesis on OKAN-II (20, 33). Although authors that used Equation (3) did not claim a physiological implication for their OKAN-II model, the choice of the equation may influence the estimate of the OKAN-I parameters. Our data showed that, in the participants showing OKAN-II, the initial velocity of OKAN-I was significantly lower for all stimuli lasting 3 min or more (i.e., when OKAN-II occurred in over 70% of the trials), than in the trials following the 30 s stimuli (i.e., when OKAN-II occurred only in 10% of the trials). A similar difference was not observed in the participants not showing OKAN-II. This suggests that OKAN-II affect the initial velocity of OKAN, as it opposes OKAN-I from the moment the light is terminated. As Equation (3) provides zero-contribution for initial OKAN-II, its use may underestimate the initial velocity of OKAN-I.

Thus, Equation (2) is the first attempt to fit OKAN-II in human data with a model compatible with the current hypothesis on the physiology underlying OKAN-I/OKAN-II interaction (33). On the other hand, while Equation (2) can be the best option to account for OKAN-II in humans without using an overly complex model (and risking overfitting), it must be kept in mind that Equation (2) does not provide an ideal fit and it may prevent delving deeply into the process generating OKAN-II or to detail its human specific features. Proper modeling of OKAN-II in humans remains challenging.

The model-based analysis failed to identify a change in the OKAN-II parameters with increasing stimulus duration. Although the validity of the fitted parameter should be considered with care, given the low *r*^2^ and the complexity of fitting OKAN-II data on humans, this finding is consistent with the area-based and velocity-based analysis. Thus, our overall results do not support the observation of Brandt et al. (20) that OKAN-II increases with stimulus duration. On the other hand, our assumptions are in line with the theoretical interpretation proposed by Brandt et al. (20) that OKAN-I and OKAN-II are two independent phenomena that sum and generate a specific pattern of OKAN reversal depending on their intensity. It must be also considered that, in comparison to their study, our model-analysis is less sensitive to the duration of the recorded data, to velocity offset and noise, but incorporates more assumptions on the two phenomena. Therefore, our data complement and improve the early estimate by Brandt et al. rather than oppose it.

Specifically, our results support the idea that OKAN observed following prolonged optokinetic stimulation in humans (i.e., earlier approach to zero and reversal of SPEV) may be described by an independent, superimposed OKAN-II (20). Such description of OKAN-II is compatible with an adaptation phenomenon that develops during the preceding OKN stimuli. The adaptation is described as a negative velocity command that is added to the OKAN-I and decays over a longer time. It is worth to note that the OKAN-II time constant estimated with this method appears similar to the one of the adaptive behaviors observed during VOR and responsible for a secondary nystagmus (34, 35). Due to this similarity, it cannot be excluded that both OKAN-II and VOR might share a common neural adaptation being responsible for both secondary responses.

This interpretation would need to be corroborated by further studies comparing the two phenomena among the same participants and, if possible, using a more detailed model as the one proposed by Furman et al. (33). It would imply that such adaptation occurs in the shared neural element, either located in the velocity storage mechanism or downward toward the motoneurons, rather than an aftereffect related to visual tracking as suggested by Chen et al. (30).

Regarding the assessment of OKAN-I, we propose that for the short testing of OKAN (up to 1 min), OKAN-II can be considered as not charged and does not impact OKAN-I.

### Limitations

Tijssen et al. suggested to perform as much as eight measurements for each condition to get a reliable estimate for the initial velocity amplitude and the time constant, because of the larger intraparticipant variability (8). Because of the strain on participants of prolonged testing, we only managed to collect two measurements for each duration, which may have led to more variability in the data.

Due to the strong variability of OKAN-I and OKAN-II in humans, the fitting with the simplified model wasn't optimal (Figure 3). With OKAN-II occurring consistently in 5 participants, the model failed to fit the data of one of them. Although this was mostly due to noise in the data and poor concentration of the participant during OKAN recording after prolonged OKN stimuli, it evidences how the weakness of the OKAN-II responses in humans may easily challenge its evaluation. To really proof if the model fits the data appropriately, more subjects as well as more trials with prolonged optokinetic stimulation should be used, although repetitive prolonged OKN trials are hard to administer in humans.

In the visual inspection of the data, we observed in some participants an offset in the end of OKAN. As we found no physiological reason to add an offset term into Equation (2) and we aimed at keeping the number of parameters low, we use a fitting equation without any offset. As a consequence, our fit was suboptimal in these participants.

As the scope of the paper was to provide a description of OKAN-II compatible with the limitations posed by its limited occurrence in humans [limited number of repetitions and low reliability of each trial (8)], we did not consider more complex models, i.e., describing the adaptation as an oscillatory behavior.

## Data Availability Statement

The datasets generated for this study are available on request to the corresponding author.

## Ethics Statement

The studies involving human participants were reviewed and approved by Cantonal Ethics Committee Zurich, Switzerland. The patients/participants provided their written informed consent to participate in this study.

## Author Contributions

JG, GB, and DS: conceptualization. JG: selection of participants, experimental measurements, and writing original draft. JG, GB, FR, and CB: data analysis. GB, FR, DS, and NF-D: review and editing. All authors: contributed to the article and approved the submitted version.

## Conflict of Interest

The authors declare that the research was conducted in the absence of any commercial or financial relationships that could be construed as a potential conflict of interest.
